# Regulation of Metabolism by an Ensemble of Different Ion Channel Types: Excitation-Secretion Coupling Mechanisms of Adipokinetic Hormone Producing Cells in *Drosophila*

**DOI:** 10.3389/fphys.2020.580618

**Published:** 2020-10-29

**Authors:** Rebecca J. Perry, Cecil J. Saunders, Jonathan M. Nelson, Michael J. Rizzo, Jason T. Braco, Erik C. Johnson

**Affiliations:** Department of Biology, Wake Forest University, Winston-Salem, NC, United States

**Keywords:** ion channel, transcriptome, metabolism, starvation, endocrine

## Abstract

Adipokinetic Hormone (AKH) is the primary insect hormone that mobilizes stored energy and is functional equivalent to mammalian glucagon. While most studies have focused on exploring the functional roles of AKH, relatively little is known about how AKH secretion is regulated. We assessed the AKH cell transcriptome and mined the data set for specific insight into the identities of different ion channels expressed in this cell lineage. We found reliable expression of multiple ion channel genes with multiple members for each ionic species. Specifically, we found significant signals for 39 of the either known or suspected ion channel genes within the *Drosophila* genome. We next performed a targeted RNAi screen aimed to identify the functional contribution of these different ion channels that may participate in excitation-secretion coupling in AKH producing cells (APCs). We assessed starvation survival, because changes in AKH signaling have previously been shown to impact starvation sensitivity. Genetic knockdown of three genes (*Ca-Beta*, *Sur*, and *sei*), in AKH producing cells caused highly significant changes (*P* < 0.001) in both male and female lifespan, and knockdown of six other genes (*Shaw*, *cac*, *Ih*, *NaCP60E*, *stj*, and *TASK6*) caused significant changes (*P* < 0.05) in only female lifespan. Specifically, the genetic knockdown of *Ca-Beta* and *Sur* led to increases in starvation lifespan, whereas the knockdown of *sei* decreased starvation survivorship. Focusing on these three strongest candidates from the behavioral screen, we assessed other AKH-dependent phenotypes. The AKH hormone is required for starvation-induced hyperactivity, and we found that these three ion channel gene knockdowns changed activity profiles and further suggest a modulatory role of these channels in AKH release. We eliminated the possibility that these genetic elements caused AKH cell lethality, and using independent methods, we verified expression of these genes in AKH cells. Collectively, these results suggest a model of AKH-cell excitability and establish an experimental framework for evaluating intrinsic mechanisms of AKH release.

## Introduction

In excitable tissues, changes in membrane potential are paramount in mediating specific aspects of cell physiology, including muscle contraction and hormonal secretion. A major molecular component that alters membrane potential are ion channels. Ion channels display considerable variation in their activation kinetics, their gating mechanisms, the specific ionic species that can pass (Hille, 2001). However, it is thought that the specific biophysics and biochemistry of the exact ion channels ultimately bestow the cell with its excitable properties. These channels shape the regular contraction cycles of cardiac muscle and determine the probabilities of formation of action potentials. In this study, we aimed to determine the ion channels present in an endocrine gland in *Drosophila*, which is paramount in regulating metabolism.

In insects, the Adipokinetic hormone (AKH) is the functional equivalent of mammalian glucagon, as it is the primary hormone that facilitates the mobilization of stored energy (Gäde and Auerswald, 2003). During periods of low circulating energy, AKH is secreted from a discrete subset of neuroendocrine cells and binds to its specific receptor (AKHR) (Park et al., 2002; Staubli et al., 2002). AKHR is expressed in the insect fat body (Grönke et al., 2007), which is the principal organ involved in fat and carbohydrate stores. AKHR activation leads to increased glycogen phosphorylase activity as well as the mobilization of stored lipids through lipase activity (Van Der Horst, 2003; Grönke et al., 2007). In *Drosophila*, AKHR is also expressed in a small subset of central neurons, where is thought to facilitate gustatory sensitivity (Inagaki et al., 2014; Jourjine et al., 2016; Yu et al., 2016), as well as in other tissues in other insects (e.g., Jedlička et al., 2016).

In *Drosophila*, targeted ablation or blockade of hormonal release of AKH-expressing neuroendocrine cells cause increased survival during starvation (Lee and Park, 2004; Isabel et al., 2005). The underlying mechanism thought to lead to lifespan extension is the accumulation of energy stores and a loss of starvation-induced hyperactivity (Lee and Park, 2004; Isabel et al., 2005). These phenotypes are also exhibited in strains that lack either the AKH hormone or its specific receptor (Bharucha et al., 2008; Braco et al., 2012; Gáliková et al., 2015) and implicate the AKH hormone as being a critical regulator of normal responses to starvation. Despite the importance of AKH as a metabolic regulator, little work has explored the physiology of AKH cells with specific regards to the mechanisms that couple nutrient sensing to changes in AKH cell excitability.

AKH expressing cells are intrinsically sensitive to changes in hemolymph sugar levels and show elevated calcium levels when trehalose levels decrease (Kim and Rulifson, 2004; Braco et al., 2012). We previously identified that the AMP-activated protein kinase (AMPK) is critical for heightened AKH secretion during low energy conditions, and the mechanism of action is the modulation of AKH cell excitability (Braco et al., 2012). Additionally, AMPK has been shown to alter the function of several different ion channel species (Evans et al., 2009). Furthermore, AKH cells are electrically excitable, display spontaneous electrical activity (Bloemen and de Vlieger, 1985), and introduction of a potassium leak channel mirrors AKH loss of function phenotypes suggesting an electrical silencing of AKH release (Kim and Rulifson, 2004; Braco et al., 2012).

Thus, we suspect that AKH release is modulated at the level of excitation-secretion coupling and hormone secretion is ultimately dependent on the specific biochemistries and biophysics of the cohort of ion channels expressed in this cell type. To investigate the potential mechanisms gating AKH secretion, we first evaluated the specific AKH cell transcriptome using a single cell RNA sequencing platform. We identified varying but consistent levels of expression of 39 different ion channel genes present in the *Drosophila* genome. We then performed a screen to identify ion channels regulating AKH release and employed RNAi elements that targeted these different ion channels genes identified in the transcriptome. Using starvation lifespan as a behavioral readout, we identify a cohort of ion channels that we suspect are critical for regulating AKH cell excitability. Paramount in this process, are the channels encoded by the *seizure*, *Ca-Beta*, and *Sur* genes. These results implicate these channels as important factors that mediate AKH hormonal release and suggest a model of AKH-cell excitation-secretion coupling.

## Results

### AKH Cells Express Multiple Ion Channel Types

To gain insight into the regulatory mechanisms that underlie AKH cell excitability and AKH hormonal release, we assessed the AKH cell transcriptome. Individual AKH cells were identified by introducing a GFP reporter prior to microdissection and RNA sequenced. The transcriptome was assessed from five replicate individuals each that had experienced 24 h of starvation and compared to animals that were fed *ad libitum*. An initial analysis of the RNA sequencing of 13 billion nucleotides corresponding to 11,456 transcripts showed no significant effects of the starvation experiments on the normalized levels of ion channel encoding genes, and consequently all replicates were pooled for further analysis.

**FIGURE 1:**
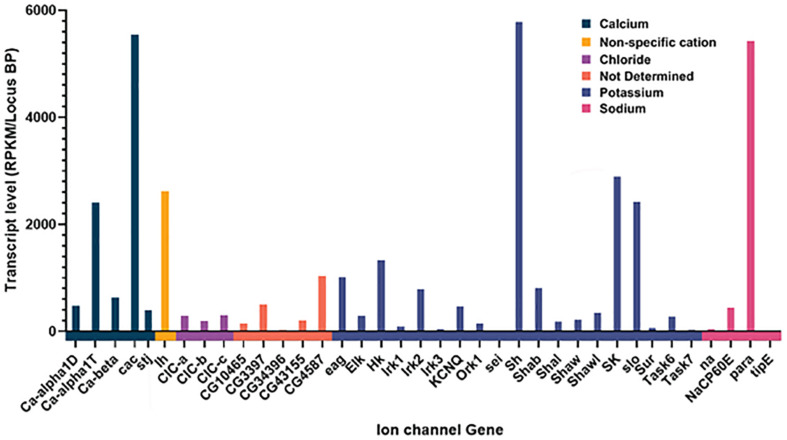
Expression of Ion channel genes in AKH cells through sc-RNAseq. Channels are grouped together by specificity and the normalized amount of transcript is plotted for each ion channel gene. Normalization was the total number of reads per kB mapped and normalized for transcript length.

Of the 47 genes that are either known or suspected to encode ion channel genes (Littleton and Ganetzky, 2000), we found reliable and consistent expression of 39 of them (Figure 1). We found expression of all the calcium channel members present in *Drosophila*, as well as every known sodium channel. Multiple potassium channel and chloride channel types were also found, and other suspected but as of yet, not molecularly determined ion channel genes. While multiple channel types were found, the relative abundance of each channel type was far from uniform, with only a handful of ion channels being highly expressed (as defined by two standard deviations from the mean expression level), and the vast majority having a consistently low level of expression. Given the molecular stability of ion channel proteins, low levels of transcript expression are expected and consistent with other transcriptome analyses in *Drosophila* (Allen et al., 2020) and other animals (Fuzik et al., 2016).

### RNAi Knockdown of Specific Ion Channels Leads to Starvation Phenotypes

Based on the transcriptome data, we next wanted to evaluate the functional significance of this ensemble of different ion channels in mediating AKH cell excitability. Ideally, direct measurements of AKH cell membrane potential would be revealing, but the anatomy of adult AKH cells makes this infeasible if not impossible with current electrophysiological techniques. We employed a genetic behavioral screen to specifically evaluate the potential contributions of different ion channel genes in modulating AKH cell physiology. This behavioral screen was based on previous observations that the genetic ablation of AKH cells or loss of function variants of the hormone and receptor all produce animals that are long-lived under a starvation paradigm (Lee and Park, 2004; Isabel et al., 2005; Bharucha et al., 2008; Gáliková et al., 2015). Furthermore, we have previously used starvation lifespan to gauge the contribution of the AMP-activated protein kinase (AMPK) as an intrinsic AKH nutrient sensor (Braco et al., 2012). We used a specific GAL4 to limit expression of a DS-RNA targeting a specific ion channel gene to only AKH-expressing cells and assessed survivorship during starvation (Lee and Park, 2004). The RNAi elements were purchased from Bloomington Stock Center and were derived from the Transgenic RNAi Project (TRiP) and has the benefit of all the elements sharing a genomic insertion site and genetic background (Perkins et al., 2015). At the time of these experiments, we were able to get and test RNAi lines for each ion channel expressed in AKH cells (Figure 1), with the exception of *Clc-A* and *Irk1*.

**FIGURE 2:**
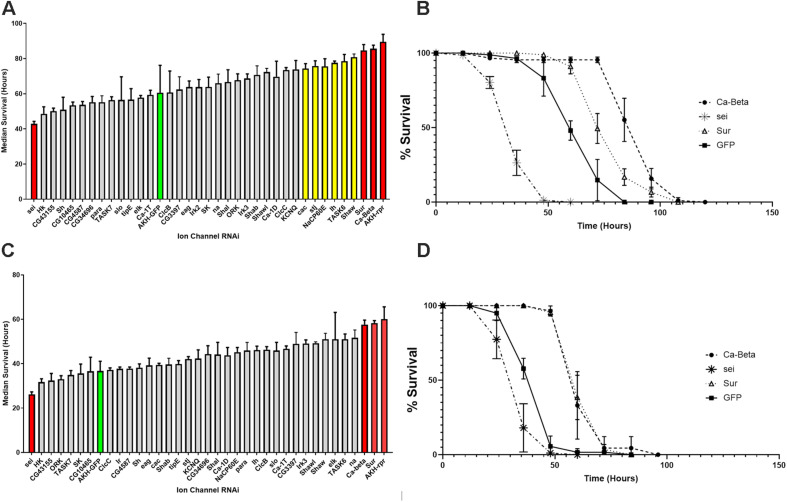
Starvation lifespan in strains with AKH-cell specific genetic knockdowns of different ion channel genes. The mean median survival was derived from three replicate vials of 30 individuals for each sex and was assessed during starvation conditions every 12 h. Mean median survival was determined for each genotype and is shown ± SEM. Bars in red denote significance levels at *P* < 0.01 and yellow bars denote significance levels at *P* < 0.05 from a One Way ANOVA and a Tukey *post-hoc* test comparing mean survival to the AKH-GFP controls (green). **(A)** Female starvation results are shown in the top panel and male starvation results are shown in the bottom panel **(B)**. Starvation lifespan curves are plotted for the three strongest candidates (Sur, Ca-Beta, and sei) with control (AKH-GFP-RNAi) for females **(C)** and males **(D)**.

We evaluated starvation longevity in progeny of the crosses introducing an RNAi element to AKH cells. Introduction of RNAi elements targeting the *Calcium Beta* (*Ca-Beta*) and the *Sulfonylurea receptor* (*Sur*) gene caused a highly significant (ANOVA, *p* < 0.001) increase in lifespan during starvation. Conversely, expression of an RNAi element targeting the *seizure (sei)* gene caused a highly significant reduction in starvation lifespan in both males and females as compared to control animals (AKH-GFP) (*P* < 0.001) (Figure 2). Notably, this GFP control line shares the same transgene insertion site as all of the RNAi elements, making it the ideal genetic control. In females, we found six other genes that significantly (*P* < 0.05) deviated from our control line and include *cac*, *stj*, *NaCP60E*, *Ih*, *TASK6*, and *Shaw* (Figure 2A). We also included a long-lived control (AKH-rpr), in which AKH cells are genetically ablated through the introduction of the pro-apoptotic gene, *reaper*, for the sake of comparison. We found that all lines were statistically different than this control variant, apart from the *Ca-beta* and *Sur* knockdowns, and this was similarly independent of sex. We independently repeated the screen with a subset of lines including all the candidates and three lines that did not differ from our AKH-GFP control line and found the same rank order of starvation sensitivity. Given that the screen yielded three candidates that were the same in both males and females, and showed the most robust differences between control lines, we focused on these three candidates. To further validate these three candidates, we obtained independently derived RNAi lines targeting these three ion channel encoding genes. We tested these lines for starvation lifespan and did not find any statistical difference between the two lines for each candidate (*P* = 0.86, *T*-Test).

**FIGURE 3:**
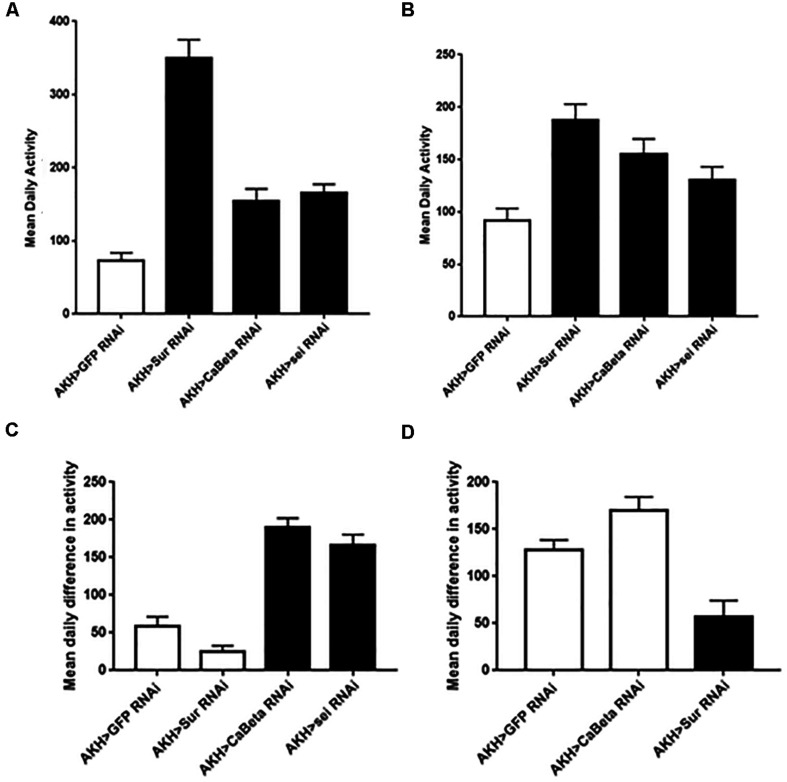
Locomotor activity in animals expressing the Sur-RNAi, sei-RNAi, and Ca-beta RNAi elements under replete and starvation conditions. Locomotor activity was monitored for 3 days with *ad libitum* food and mean daily activity (beam breaks for 24 h) was assessed for females **(A)** and males **(C)**. Black bars denote significant differences (*P* < 0.05) from control animals (One Way ANOVA). Locomotor activity during starvation conditions was also assessed in females **(B)** and in males **(D)**. The hypersensitivity of sei-RNAi to starvation precluded measuring daily activity during starvation). Black bars denote significant differences (*P* < 0.05) from control animals (One Way ANOVA).

Another AKH-dependent phenotype is the requirement of this hormone for starvation-induced hyperactivity (Lee and Park, 2004; Isabel et al., 2005; Bharucha et al., 2008; Braco et al., 2012; Gáliková et al., 2015). Specifically, the loss of the AKH receptor, the AKH hormone, or AKH expressing cells all cause the absence of heightened activity that accompanies starvation in normal flies. This behavior is thought to facilitate heightened foraging (Johnson, 2017). Consequently, we hypothesized that if these three ion channel genes are altering the excitability of AKH cells, then these genetic manipulations should manifest as changes in locomotor activity profiles. We found that locomotor activity under replete conditions was heightened in animals expressing any of these three ion channel RNAi elements in both males and females (*P* < 0.001) (Figure 3). Next, we asked whether locomotor profiles change in response to starvation in animals with altered ion channel function? In males, we found that RNAi knockdown of *Sur* and *Ca-Beta* led to significantly reduced changes in starvation-locomotion compared to wildtype. We excluded the *sei* RNAi males, because a significant number of males would have died during our window of analysis. In females, we found that there was no increase in locomotion in wildtype and *Sur* RNAi females. Knockdown of *seizure* and *Ca-beta* caused elevated locomotor responses to starvation (Figure 3).

**FIGURE 4:**
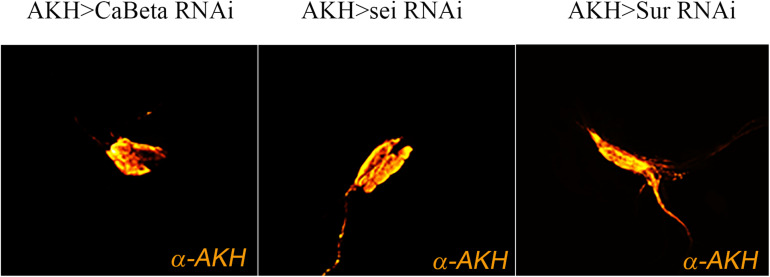
Expression of ion channel RNAi elements do not change AKH cell viability. Expression of ion channel RNAi’s does not impair cell viability or AKH synthesis. Representative images of adult AKH cells expressing Ca-Beta-RNAi **(left)**, sei-RNAi **(middle)**, and Sur-RNAi **(right)** counterstained with α-AKH.

One potential explanation for the behavioral phenotypes is that these RNAi elements may adversely affect AKH cell viability or impair hormone synthesis. To evaluate potential AKH cell pathology associated with RNAi element expression, we employed a specific antibody against AKH to assess cell survival and hormone expression. We found that in all genotypes, AKH cell viability and minimally, synthesis of the AKH precursor were unaffected (Figure 4). These results indicate that the behavioral phenotypes are not likely to be caused by developmental defects, AKH cell lethality, or the lack of AKH hormone expression caused by RNAi introduction. We next sought to confirm the transcriptome and behavioral data and independently assessed whether these ion channel genes are expressed in this cell lineage. We employed single-cell RT-PCR on AKH cells and were able to specifically amplify all three of these genes. Amplicons were detected from AKH, Ca-Beta, sei, and Sur transcripts and were of the correct size and were sequence verified (data not shown).

## Discussion

Here, we present evidence that multiple ion channel genes are expressed in the neuroendocrine cells that release the Adipokinetic Hormone. Multiple ion channel transcripts were identified through an analysis of the AKH-cell specific transcriptome. We then employed a genetic approach to determine the overall contribution of these ion channels in modulating AKH secretion through the examination of AKH related starvation phenotypes. We identified that the genetic knockdown of three ion channel encoding genes strongly impacted starvation survival and found several other genes whose impacts were significant but modest in comparison. We also examined locomotor activity from animals expressing an RNAi targeting each of these three ion channel genes whose AKH cell specific knockdown highly impacted starvation survival. In each case, changes in locomotor activity were present either in the replete or starvation state. Lastly, we verified the knockdown of these genes did not cause AKH cell lethality, and we employed independent measures to confirm the AKH cell specific expression of these genes.

One question that arises from our transcriptome results: Why are there so many different channels? We note that most of the ion channel genes within the *Drosophila* genome are expressed in this secretory tissue. However, we note that single cell (sc) transcriptomes of individual neurons also show a diversity of ion channels present (Fuzik et al., 2016; Northcutt et al., 2019). While many of these sc-RNA studies attempt to elucidate potential genetic heterogeneity of either a brain area or groups of neurons that share a transmitter phenotype, it is clear that a meta- analysis of sc-transcriptomes from mammalian and invertebrate neuronal tissue show multiple channel types present in an individual neuron. We also cannot rule out the possibility that some of our transcriptome samples stem from synaptic terminals projecting to AKH cells that may express a diversity of ion channels. Nonetheless, our transcriptome analysis represents a first requisite step in gauging the complement of genes expressed in APCs and is consistent with other neuronal sc-transcriptomes (Fuzik et al., 2016; Northcutt et al., 2019) and mammalian pancreatic sc-transcriptomes (Muraro et al., 2016).

**FIGURE 5:**
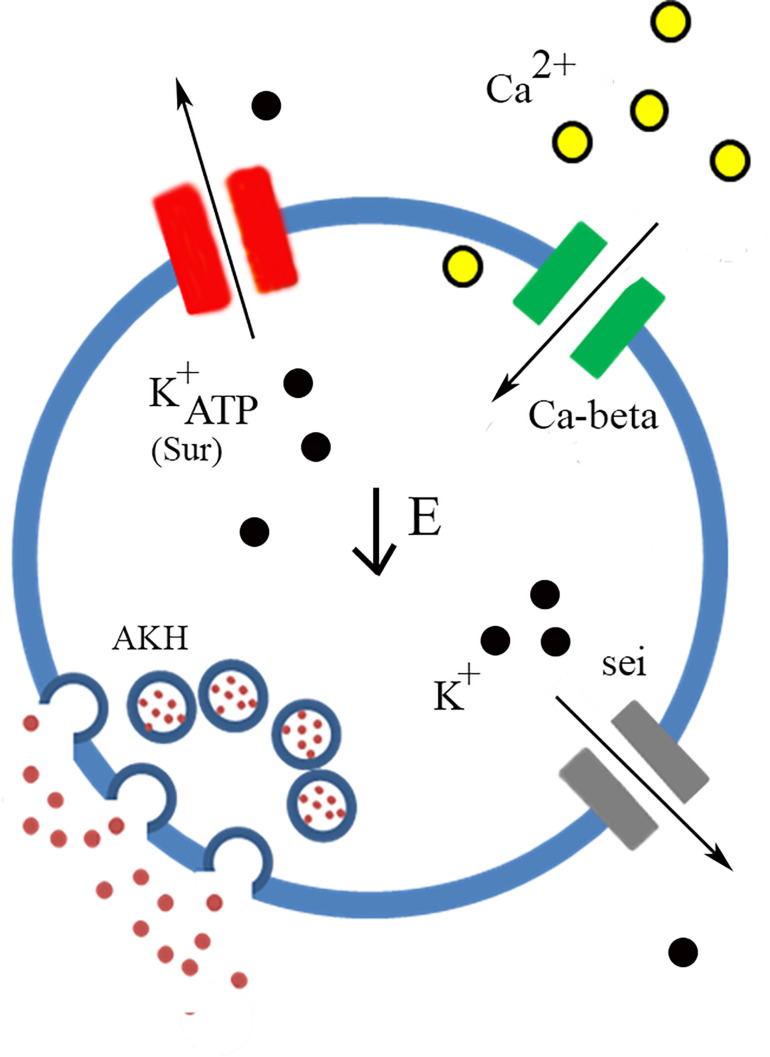
Model of AKH cell excitability. Low Energy (center) is the trigger that facilitates AKH release. Nutrient sensing is likely to occur through the ATP gating of K^+^_*ATP*_ channels of which *Sur* is an essential component. Calcium enters through the cell through calcium channels and the *Ca-beta* subunit is likely a critical component of functional calcium channels present in AKH cells. Potassium channels, likely containing *sei* subunits, also participate in the regulation of AKH cell excitability and the subsequent secretion of the hormone.

Following our transcriptome analysis, we performed a targeted RNAi screen using AKH-dependent behavioral phenotype and identified three genes (*Ca-Beta, Sur*, and *seizure*) that are strong candidates in the regulation excitation-secretion coupling in this cell lineage (Figure 5). As in the case of any behavioral screen, negatives are not necessarily informative, although we maintain that some of the reasons that we failed to identify other strong candidates may reflect biological vs. technical reasons. Unfortunately, the technical limitations of the single-cell RT PCR and the expense of performing a transcriptome on each genetic variant make quantification of transcript levels of the target genes infeasible. However, we do note that all these lines share a genetic insertion site (Perkins et al., 2015), suggesting that if there are differences in the lines, those difference map to the specific DS-RNA species. Ion channels are known to form heteromultimeric complexes that often consist of different subunits (Cai, 2008), and multimeric assembly of ion channels potentially explains both the observation of many different channel types present via the transcriptome analysis and that relatively few manipulations lead to AKH phenotypes. Furthermore, the fact that there are many different ion channels that pass the same ion suggests that there could be considerable functional redundancy. For example, it is interesting that we found a strong starvation phenotype from knockdown of a regulatory subunit of a calcium channel (*Ca-Beta*) and despite the observation of all known pore-forming calcium channel genes being present in AKH cells via the transcriptome, knockdown of those produced largely no effect. We do note that the knockdown of *cac*, which is a pore forming calcium channel subunit (Smith et al., 1998) produced a significant effect on starvation, albeit weaker in comparison to the Ca-Beta knockdowns. We speculate that this observation may reflect the presence of multiple different functional calcium channel types in AKH cells, and that *Ca-beta*, being the sole regulatory subunit, is a critical component that regulates disparate calcium channels. Its worthy to note that, in mammals, Ca-Beta subunits show diverse functions including channel trafficking, activation/inactivation kinetics, and setting voltage dependence (Buraei and Yang, 2013). We also note that our transcriptome showed a wide variation in ion channel expression levels, and we note that the most robust phenotypes targeted ion channel genes that were typically lower in expression levels, and that were novel subtypes. Furthermore, we did observe weaker starvation phenotypes in a number of different ion channel genes (*Shaw*, *cac*, *Ih*, *NaCP60E*, *stj*, and *TASK6*), but only in females. We suspect that the behavioral output (starvation lifespan) has greater resolution in females as they live considerably longer during this challenge, as opposed to an inherent dimorphism mapping to AKH cells.

An important result of the behavioral analysis is the further confirmation of the *Sur* gene as being a critical factor that regulates AKH secretion. Specifically, *Sur* is one component of the functional K^+^_*ATP*_ channel (Nichols, 2006) and had been previously implicated in being expressed in larval AKH expressing cells (Kim and Rulifson, 2004). Notably, the other component of the K^+^_*ATP*_ channel include an inward rectifying potassium channel, and in *Drosophila* there are three distinct genes that encode channels in this subfamily (Littleton and Ganetzky, 2000). None of these IRK channels produced a behavioral phenotype which suggests that these components of K^+^_*ATP*_ channels are functionally redundant. The significance of this finding is that K^+^_*ATP*_ channels are, eponymously, gated by intracellular energy stores, ATP. Consequently, this channel may function as a direct link to energy status and AKH release. Of further interest is the observation that this channel type appears to gate glucagon and insulin release from pancreatic alpha and beta cells, respectively (Göpel et al., 2000; Rorsman et al., 2014). Pharmacological studies in *Drosophila* using a K^+^_*ATP*_ agonist, tolbutamide, produced metabolic phenotypes that are consistent with previous studies and our genetic interdiction of K^+^_*ATP*_ channel function (Kim and Rulifson, 2004). Mechanistically, we predict that the loss of *Sur* likely prevents AKH cells from coupling low extracellular energy to changes in membrane potential, leading to an overall reduction in AKH signaling. Of note, we previously identified that AMPK is critical for normal AKH cell function, and acts as an intrinsic nutrient sensor (Braco et al., 2012). We also determined that AMPK activation leads to enhanced AKH cell secretion and is most likely at the level of excitation-secretion coupling. Our results and those presented in the literature suggest that a key future experiment is to test whether these two nutrient sensors present in AKH cells act independently or in concert.

Mutations in *sei* have previously shown to lead to increased spontaneous depolarization in other tissues in a temperature sensitive manner (Zheng et al., 2014), consistent with the idea that the normal potassium conductance passed by this channel would lead to hyperpolarization. Genetic knockdown of *sei* showed a significant reduction in lifespan and increased locomotion under replete and starvation conditions. These phenotypes suggest an increased level of AKH secretion. Of interest, mammalian pancreatic alpha cells express hERG channels, a homolog of *sei*, which regulates resting membrane potential (Qiu et al., 2016), and acute pharmacologic blockade of ERG channels shows an overall reduction in glucagon secretion (Qiu et al., 2016). The finding of *seizure* channels as a regulator of AKH secretion suggests that there may be significant parallels in the regulatory components of glucagon secretion and AKH cells. Interestingly, many of the channels we found essential for AKH secretion are similar to the channels regulating glucagon secretion (Rorsman et al., 2014).

AKH cells are a critical locus for integrating sensory information regarding energy status and facilitating appropriate behavioral and physiological responses (Johnson, 2017). Specifically, these cells integrate intracellular status of energy within the context of other hormones to coordinate an appropriate behavioral and physiological response to nutrient depletion. An important component of these responses is that they are fundamentally graded (i.e., an hour of restricted food availability is different than 24 h of starvation). Inspection of AKH loss of function phenotypes clearly shows that AKH is required for starvation-induced hyperactivity, although the locomotor phenotypes we observed in AKH cell specific knockdown of these ion channels suggest a more complex role of AKH in determination of total locomotor activity. Indeed, AKH has been shown to impact different aspects of locomotion in a wide variety of insects that appear to be separate and distinct from nutrient limitations (e.g., Kodrík, 2008). Nonetheless, as we suspect that AKH secretion is being decoupled from nutrient sensing in these genetic variants targeted ion channel function, we observed changes in basal (replete) locomotion as we expected. The interaction between abnormal secretion events and starvation suggest a more complex role of AKH in these behaviors. We acknowledge that lifespan under starvation is influenced by multiple physiological setpoints and is a rather conservative measure to use to identify candidate molecules that regulate AKH cell physiology. Likewise, the profound impact on locomotor activity observed in variants that are complete loss of AKH functions, may not be expected to be observed in genetic manipulations that modulate AKH cell activity. Consequently, while the changes in locomotor activity in AKH deficient animals may lead to heightened starvation survival, the connections between starvation survival and locomotor activity are likely to be more complicated than anticipated.

Given these behavioral phenotypes, it is reasonable to ask whether these genetic perturbations of ion channel function negatively impact APC physiology vs. a specific secretion phenotype. We used immunohistochemistry to evaluate the general health of APCs and documented that they were present and, minimally expressing the AKH precursor. The next requisite step would be direct measurements of APC excitability using electrophysiological methods. Indirect measures of AKH concentrations, either in APCs or in the hemolymph clearly correlate with secretion profiles but are not solely measuring secretion. We, and others have previously used immunocytochemistry to evaluate AKH concentrations within APCs (Braco et al., 2012; Kim and Neufeld, 2015) or in the hemolymph (Oh et al., 2019). Cellular AKH levels are dependent on secretion rates, but also the degree to which the hormone is transcribed and processed. Circulating AKH levels are subject to peptidase degradation and receptor binding. We have used secretion reporters previously to document that AKH cell secretion is dependent upon AMPK activation, and while this direct measure of cellular secretion is preferable to indirect measures outlines above, such reporters are better suited to measure differentials (Levitan and Shakiryanova, 2010) and would not be useful in this study to measure changes in the predicted spontaneous peptide release as a consequence of altered APC excitability. We are attempting to measure parameters of APC excitability using electrophysiological methods as such information will provide critical insight into the precise mechanisms of altered APC function.

Nonetheless, we maintain that there is most probable an ensemble of ion channels that regulate excitation-secretion of this metabolic hormone and any other potential cotransmitters, and that we have identified a select few channels, whose functional importance is larger than others. Consequently, our screen informs future studies that directly assess AKH cell excitability and has the potential to establish parallel mechanisms of nutrient sensing to hormone release across wide phylogenetic distances. For example, our screen informs experiments aimed at direct electrophysiological assessment of APCs, which will be able to resolve the specific details regarding APC excitability and hormonal release.

## Materials and Methods

### Drosophila Stock and Husbandry

All flies were maintained in an incubator maintained at 25°C and under a 12:12 LD cycle. Flies were cultured on a standard molasses-malt-cornmeal-agar-yeast medium and housed in uncrowded conditions. All stocks were purchased from Bloomington stock center and are listed in Table 1.

**TABLE 1:** List of ion channel RNAi elements used in this study.

Gene name	Bloomington stock number(s)	Ion selectivity
*Ca-alpha 1D*	33413	Calcium
*Ca-alpha 1T*	39029	Calcium
*Ca-beta*	29575, 43292	Calcium
*cacophony (cac)*	27244	Calcium
*CG10465*	26002	Suspected potassium
*CG3397*	44115	Suspected anion
*CG34696*	26011	Suspected potassium
*CG43155*	27033	Suspected potassium
*CG4587*	25893	Suspected anion
*Clc-B*	25826	Chloride
*Clc-C*	27034	Chloride
*eag*	31675	Potassium
*elk*	25821	Potassium
*Hk*	28330	Potassium
*Irk2*	41981	Potassium
*Irk3*	26720	Potassium
*KCNQ*	27252	Potassium
*Ih*	29574	Cation
*na*	25808	Sodium
*NaCp60E*	26012	Sodium
*Ork1*	25885	Potassium
*para*	31471	Sodium
*sei*	31681, 31682	Potassium
*Sh*	31680	Potassium
*Shab*	41999	Potassium
*Shal*	31879	Potassium
*Shaw*	28346	Potassium
*Shawl*	25819	Potassium
*SK*	27238	Calcium-activated Potassium
*slo*	26247	Calcium-activated potassium
*stj*	25807	Potassium
*Sur*	36087, 67246	Potassium
*TASK6*	28015	Potassium
*TASK7*	27264	Potassium
*tipE*	26249	Sodium
*AKH-GAL4*	25864	NA
*UAS-GFP*	35786	NA
*UAS-rpr*	5823	NA

**Each ion channel gene name along with the corresponding Bloomington Stock number and the suspected or predicted ion channel selectivity is listed. Ion channel selectivity information is based on Littleton and Ganetzky (2000).**

### RNA Sequencing

AKH cells expressing GFP under the AKH promoter (12 per animal) were microdissected in HL-3 solution containing Triton X 100 (Braco et al., 2012) and aspirated into a glass pipette, which was placed in a PCR tube and flash frozen in an ethanol-dry ice bath. The tubes were stored at −80°C for no longer than 3 weeks while 10 samples were prepared from 5 fed and 5 starved female flies aged 3–10 days. On the day of RNA amplification, the contents of the pcr tubes were centrifuged and the RNA from these samples was amplified in parallel with the Arcturus RiboAmp HS PLUS Kit by following the manufacturers protocol (KIT0505, Thermo Fisher Scientific). RNA libraries were then prepared using the Kapa Stranded mRNA-Seq library prep kit and 50 bp single end sequencing was performed on an Illumina HiSeq 4000 at Duke Center for Genomic and Computational Biology (Durham, NC). This data is available at the NCBI Sequence Read Archive under project number PRJNA642982.

The raw reads were filtered using Trimmomatic v0.36 to remove Illumina adaptors, leading or trailing bases below a quality score of 3, 4-base sliding window average quality below 15 and reads less than 36 bp long. Filtered reads were aligned to *Drosophila melanogaster* genome BDGP6.22 using star v2.5 (Dobin et al., 2013) and a count table generated from coordinate sorted BAM files using summarize overlaps from the biocondutor package GenomicAlignments (Lawrence et al., 2013). We identified genes differentially expressed by starvation using the Bioconductor package DESeq2 (Love et al., 2014) but no ion channels were differentially expressed under starvation conditions (adjusted *p* > 0.05). The source code for this analysis is available at github (Saunders, 2020).

### Lifespan Measurements

We placed 30, 3–5 day old mated flies (males and females housed separately) in vials with a two percent agar solution to starve the animals (Zhao et al., 2010). We assessed percent survival of at least three replicate vials twice daily. For each vial, we assessed the median survival for the treatment and data were pooled to estimate a mean median survival and then employed a one-way ANOVA with *post-hoc* Tukey’s comparison for differences between genotypes.

### Locomotor Measurements

Locomotor activity was monitored with a TriKinetics Locomotor Population Monitor (Waltham, MA) on the aggregate population of 30, 3–5 day old flies (Zhao et al., 2010). Each vial was considered a replicate and 3–4 replicates per condition were run. Flies were housed in a 12:12 LD cycle for 3 days prior to the experiments. Flies were transferred to a vial containing starvation or normal medium at ZT0. Total beam counts were monitored continuously through an automated system for 48 h. We determined the amount of activity during starvation relative to the activity of fed conditions for the same time period (Zhao et al., 2010; Braco et al., 2012).

### AKH Cell Imaging and Immunocytochemistry

Adult progeny from flies carrying the *AKH-GAL4* transgene crossed to the UAS-RNAi lines were dissected. Brains were fixed in a 4% Paraformaldehyde/7% picric acid fixative for 1 h at room temperature and washed six times with phosphate buffered saline (PBS) containing Triton-X 100. A 1:1,000 dilution of polyclonal anti-AKH (Brown and Lea, 1988) was incubated overnight at 4°C. Brains were washed and a Cy-3 conjugated anti-rabbit secondary antibody was applied overnight at 1:1,000 dilution. Tissues were then mounted and viewed on a Zeiss 710 confocal microscope. Images were collected using a 40X 0.95NA objective and 561 laser line, Z stack images were taken and collapsed using maximum intensity projections. Microscope settings were constant between images and adjusted post imaging for contrast and brightness.

### Single Cell RT-PCR

Individual AKH cells expressing GFP were microdissected and stored in Trizol. A Single-cell RT- PCR kit from Qiagen was used to make cDNA and amplify using gene specific primers, and methods are described in detail in Braco et al. (2012). The primers were designed to flank intronic sequences and were as follows:

*CaBeta*: (forward): ATACAATCAATCATCCGTCACAAC

(reverse): TTGATCAAACGCTGTAAAACCTTA

*Sur* : (forward) TTCAAAAGTTCTACAGGTGCTCAG

(reverse): AAGGATTTCGGTGAATCTAGTCTG

*sei*: (forward): AGGATCCCAATGACATGATTACTT

(reverse): AATTGGATCGGAGTTGATGTATTT

## Data Availability Statement

The datasets generated for this study can be found in the online repositories. The names of the repository/repositories and accession number(s) can be found below: https://www.ncbi.nlm.nih.gov/, PRJNA642982.

## Author Contributions

CS and EJ: design of experiments and analyzing data. CS, RP, JN, MR, and JB: performing experiments. EJ: drafting the manuscript. All authors contributed to the article and approved the submitted version.

## Conflict of Interest

The authors declare that the research was conducted in the absence of any commercial or financial relationships that could be construed as a potential conflict of interest.
